# Modulating Cortico-cortical Networks with Transcranial Alternating Current Stimulation: A Minireview

**DOI:** 10.1298/ptr.R0035

**Published:** 2025-03-10

**Authors:** Ryoki SASAKI

**Affiliations:** Graduate Course of Health and Social Work, Kanagawa University of Human Services, Japan

**Keywords:** Cortico-cortical networks, Transcranial alternating current stimulation, Neural entrainment, Brain oscillations, Functional connectivity, Neurorehabilitation

## Abstract

Advancements in brain imaging and analytical methods have demonstrated that behavior arises from the coordinated activity of multiple brain regions within cortico-cortical networks. Transcranial alternating current stimulation (tACS), a noninvasive brain stimulation (NIBS) technique, applies weak sinusoidal alternating currents to specific brain regions using scalp-mounted electrodes. Traditionally, tACS has been used to target single brain regions to enhance functions such as motor, sensory, and cognitive abilities. However, recent findings indicate its potential for simultaneously stimulating 2 brain regions, thereby modulating cortico-cortical network strength through neural entrainment—where brain oscillations synchronize with external rhythmic stimuli. Despite this potential, tACS applications remain primarily focused on individual brain regions. Given that behavior stems from dynamic interactions within cortico-cortical networks rather than isolated regions, this minireview explores the role of these networks in shaping behavior through functional connectivity as identified by neuroimaging. It also provides an in-depth analysis of tACS as a tool for modifying cortico-cortical networks via neural entrainment, offering promising applications in neurorehabilitation for brain disorders linked to network dysfunction. This highlights tACS as a novel approach for targeted modulation of cortico-cortical networks, distinguishing it from traditional NIBS techniques.

## Introduction

Advances in brain imaging and analytical methods have shown that behavior arises from collaborative processing among multiple brain regions rather than the activity of any single region alone^1–3)^. This processing is commonly assessed using functional connectivity (FC) through techniques such as electroencephalography (EEG)^4)^, magnetoencephalography (MEG)^5)^, and functional magnetic resonance imaging (fMRI)^6)^. FC analysis provides an advanced approach to measuring the strength of cortico-cortical networks by estimating phase synchronization or statistical correlations between oscillatory brain signals in spatially distinct regions^5,6)^. These interactions are critical for integrating neural information across brain regions simultaneously and are associated with behavior within specific cortico-cortical networks^1–3)^. This evidence indicates that modulating cortico-cortical network strength, in harmony with the brain’s natural processing, could effectively enhance behavior.

Various noninvasive brain stimulation (NIBS) methods, such as transcranial direct current stimulation^7)^, repetitive transcranial magnetic stimulation^8)^, and paired associative stimulation^9)^, have been developed to induce neuroplastic changes and show promise for enhancing brain functions in neurorehabilitation. Transcranial alternating current stimulation (tACS), another NIBS technique, delivers a weak sinusoidal electrical current (typically 1.0–2.0 mA) to a targeted brain region through the scalp using 2 or more electrodes^10–12)^. Like other NIBS techniques, tACS is often applied to individual brain regions. Numerous studies have focused on the effects of tACS on single regions, particularly the primary motor cortex (M1), where stimulation is typically applied for 10–20 min. These studies have demonstrated the ability of tACS to induce neuroplastic changes lasting for several tens of minutes^10,13,14)^. These neuroplastic changes are believed to be mediated by N-methyl-d-aspartate receptor activity^10)^. In addition to motor function, tACS can affect sensory and cognitive functions depending on the targeted brain region, such as the primary somatosensory cortex (S1) and the prefrontal cortex, respectively^15,16)^. As a result, tACS is considered a promising NIBS technique with potential applications in neurorehabilitation for enhancing various brain functions. However, NIBS techniques targeting single brain regions have recently faced significant criticism, with concerns about their weak and highly variable effectiveness^17–19)^.

**Fig. 1.**
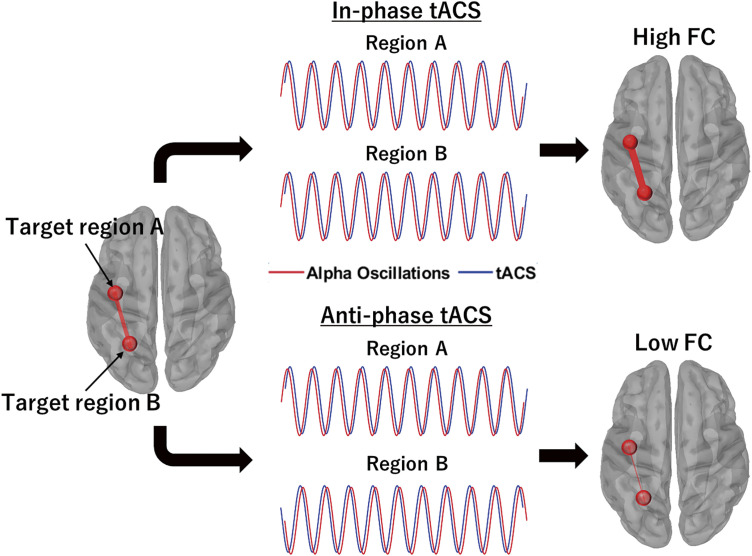
Schematic of modulating a specific cortico-cortical network using in-phase and anti-phase tACS at the alpha band (10 Hz) In-phase (0°) tACS applied to 2 target regions, A and B, may enhance their interaction through neural entrainment, leading to phase synchronization between the alpha oscillations (10 Hz) and alpha-tACS. Here, the phase difference between the 2 regions is consistently 0°. By contrast, anti-phase (180°) tACS could disrupt these interactions, potentially reducing synchronization and causing phase desynchronization between the regions, with the phase difference maintained at 180°. This model combines the principles of neural entrainment and FC. The alpha oscillations are depicted as an ideal sine wave with a constant amplitude for illustrative purposes. FC, functional connectivity; tACS, transcranial alternating current stimulation

Unlike other NIBS techniques, tACS can entrain brain oscillations at specific stimulation frequencies^20,21)^, enabling interaction with rhythmic neuronal activity^22)^. This process involves a neural population capable of generating rhythmic activity (e.g., delta, theta, alpha, beta, and gamma oscillations) at a target frequency and aligning its intrinsic activity with the phase of external stimulation^11,21)^. This phenomenon, known as “neural entrainment,” has been demonstrated in EEG and MEG studies^22–24)^ as well as in animal research^21,25,26)^. Leveraging this property, tACS has typically been applied at the same frequency as the brain rhythm observed in a specific region associated with a particular function^10,27)^. The goal is to modulate neural activity by synchronizing the brain’s endogenous oscillations with external stimulation, ensuring alignment between the phases of neural activity and tACS. This distinctive property has significantly enhanced our understanding of how the phase and power of brain oscillations influence cortical excitability and behavior. For instance, online beta-tACS at 20 Hz over M1 induces phase-dependent alterations in motor-evoked potentials elicited by transcranial magnetic stimulation^28)^. Similarly, alpha-tACS at approximately 10 Hz over the parieto-occipital region preferentially increases alpha oscillation power^29)^. If neural entrainment proves reliable, tACS could be applied to specific cortico-cortical networks to modulate their strength. In particular, applying in-phase tACS to 2 target brain regions may enhance the FC between them through neural entrainment (Fig. 1). This effect occurs because brain oscillations in each target region would theoretically synchronize with the phase of the external stimulus at the same frequency, thereby strengthening cortico-cortical interactions. Conversely, anti-phase tACS may disrupt these interactions, potentially reducing synchronization between the regions (Fig. 1). Modulating cortico-cortical interactions—whether by enhancing or diminishing them—could be more important than targeting individual regions, as behavior arises from the coordinated activity of cortico- cortical networks rather than isolated regions^1,30)^. Despite this potential, tACS is still predominantly applied to individual brain regions.

Several review articles on tACS have been published, primarily focusing on the effects of tACS on specific functions such as motor and cognitive aspects^31,32)^. and on the mechanisms of neuroplastic changes and neural entrainment^20,33)^. These reviews have provided valuable insights into the application and effectiveness of tACS across various domains. However, the impact of tACS-induced neural entrainment on cortico-cortical networks has not been thoroughly addressed in these reviews. Specifically, there is a need for a more detailed examination of how tACS can be systematically applied to modulate functional interactions between different cortical regions and how these modulations can lead to functional improvements in behavior. Understanding the precise conditions under which tACS can enhance cortico-cortical networks is crucial for advancing both theoretical knowledge and practical applications in neuromodulation. This minireview highlights the importance of cortico-cortical networks in shaping behavior and offers a detailed overview of the potential of tACS to modulate these networks, based on a comprehensive review of the current literature.

## Functional Role of Cortico-Cortical Networks in Behavior

Before exploring the effects of tACS on cortico-cortical networks, we first summarize methods for assessing cortico-cortical interactions through FC analysis. While various FC analyses are available^34)^, we focus primarily on phase- and amplitude-based connectivity measures, as these are commonly used in neuroimaging research^1,2,35,36)^. The phase-locking value (PLV), a method for assessing phase synchronization, measures the length of the average vector formed by multiple unit vectors, where each vector’s phase angle represents the phase difference between 2 time series^36)^. If the phase differences between the 2 signals are evenly distributed, the length of the average vector will be small, indicating low FC. Conversely, if the phase differences are nonuniformly distributed, the length of the average vector will be large, indicating a high FC value. By contrast, the amplitude envelope correlation (AEC), a method for assessing amplitude correlation, measures the coupling between 2 time series by estimating Pearson’s correlation between their amplitude envelopes^5,6)^. Therefore, the strength of FC in AEC is represented by Pearson’s correlation coefficient. Both PLV and AEC offer valuable insights into how multiple brain regions interact during rest or specific tasks.

These methods have been used to explore the functional role of cortico-cortical networks in specific performances, such as motor^2)^, somatosensory^1)^, and cognitive^37)^ skills. For example, our MEG study found that resting-state FC between the S1—superior parietal lobule, S1—angular gyrus, and S1—superior temporal gyrus was linked to tactile 2-point discrimination thresholds^1)^. In a follow-up MEG study, we discovered that resting-state FC between the S1—superior parietal lobule and S1—parieto-occipital sulcus was associated with tactile grating orientation thresholds^30)^. Other MEG studies have reported resting-state FC involving networks like the M1—posterior parietal cortex, M1—superior temporal gyrus, and M1—cerebellum, which are related to motor performance^2,38)^. This growing body of evidence suggests that neural correlations between specific brain regions, rather than isolated regions alone, are essential for behavioral processing. As a result, targeting and modulating specific cortico-cortical networks linked to particular behaviors may offer an effective approach to enhancing behavioral performance.

## Can a Weak Electrical Current Truly Affect Brain Oscillations?

Although tACS has the potential to modulate cortico-cortical networks, its actual impact on brain oscillations is still debated. The common belief is that weak alternating currents applied to the cortex can directly influence the membrane potential of a large population of neurons, leading to neural entrainment^33)^. However, some argue that tACS may not primarily modulate cortical activity but rather affect other factors. Studies have shown that most of the electrical current is attenuated by the skin and skull, with only a small fraction reaching the brain^39,40)^. While animal studies have demonstrated that electrical fields as low as 1 V/m can influence membrane potentials^39,40)^, the electrical field generated by tACS in human target regions is typically below this threshold^27,41)^. Recent findings suggest that the effects of tACS may primarily result from the transcutaneous stimulation of peripheral nerves on the scalp^39)^. This raises the following question: Does this mean that tACS is ineffective at directly modulating brain oscillations and cortical excitability? While there is no clear answer, some studies have shown positive results, indicating that tACS can modulate brain oscillations and cortical excitability with electrical fields below 1 V/m. Contrary to earlier research, both human and animal studies suggest that even an alternating current as low as 0.2 V/m can influence membrane potentials^29,39)^. Additionally, peripheral models have not successfully accounted for the observed increase in oscillatory power following tACS^29)^. Given these findings, it remains plausible that tACS directly affects brain activity. However, further research is needed to determine the optimal electric field strength required to achieve this effect in humans.

Although there is no universally agreed-upon optimal electric field strength for inducing neural entrainment, 0.2 V/m is often regarded as the minimum threshold in human studies. Before conducting experiments, simulations are recommended to estimate the electric field strength in the target brain region based on the chosen tACS parameters and electrode montage. SimNIBS, a widely used open-source software, allows for simulations across different parameter settings and electrode configurations (https://simnibs.github.io/simnibs/build/html/index.html)^42)^. This simulation typically uses a standard MRI template; however, individual neuroanatomical differences can cause significant variability in electric field strength^29)^, potentially affecting the efficacy of tACS across individuals. To address this, it is recommended to tailor the tACS parameters and electrode montage for each subject using SimNIBS along with personalized MRI data. The software’s MRI segmentation capabilities allow the creation of a customized head model^42)^, providing more accurate simulations. For advanced applications, SimNIBS also offers optimized settings for electrode montage and intensity to achieve the desired electrical field strength^43)^. An individualized approach is crucial for maximizing tACS efficacy; by incorporating personalized MRI data and simulations, anatomical variations affecting the electric field distribution are considered, ensuring that tACS settings are precisely tailored for each subject. This personalized approach can enhance the precision and effectiveness of tACS, helping to advance our understanding of neural entrainment mechanisms.

## tACS Targeting Specific Cortico-Cortical Networks

A comprehensive literature search was conducted on October 7, 2024, using the PubMed database to identify studies published between January 1, 2000, and October 7, 2024. The search was limited to studies involving “Humans,” published in “English,” and focusing on “Adults (19+ years)” using the following search terms: [(“transcranial alternating current stimulation”) AND (“functional connectivity”)] (Appendix 1). The selected studies had to meet the following criteria: (1) peer-reviewed original research in English, (2) conducted on adult humans (>18 years), (3) involving tACS targeting cortico-cortical networks (i.e., 2 brain regions), (4) using FC analysis via EEG/MEG, (5) assessing FC before and during or after the intervention, and (6) employing the same tACS frequency band for FC analysis. One reviewer (R.S.) conducted the initial screening of titles and abstracts. The full texts of potentially relevant articles were then assessed for eligibility. After removing duplicates, the search identified a total of 337 studies. Based on the selection criteria, 12 full-text articles were reviewed, and 6 were excluded for the following reasons: the network targeted by tACS and the assessed FC network did not match (*N* = 2); FC was not assessed (*N* = 1); FC changes between pre-intervention and during/post-intervention were not assessed (*N* = 1); tACS was applied independently to M1 and the cerebellum (*N* = 1); and the target regions varied between participants (*N* = 1).

Table 1 provides a summary of the participant numbers and methodological characteristics, such as tACS parameters, target regions, and electrode montages, in the included studies. tACS was applied to 2 regions simultaneously using either in-phase or anti-phase stimulation. The stimulus parameters varied across studies, including duration (15–20 min), frequency (6–40 Hz), and intensity (1–4 mA). The electrode montages also differed, with variations in electrode size (1.13–100 cm^2^) and the number of electrodes (2–10). The target regions in these studies included (1) visual cortices, (2) frontal–parietal regions, (3) frontal and parieto-occipital regions, (4) posterior medial frontal cortex–lateral prefrontal cortex, and (5) inferior frontal gyrus–pre-supplementary motor region. Five studies used EEG to assess FC at the sensor level, while one study used MEG at the source level. The results were mixed, with some studies reporting increases or decreases in FC under various tACS parameters, while others found no significant changes (Table 1). Three of these studies assessed behavioral changes using different tasks: working memory^44)^, visual attention^45)^, and stop signal reaction time^46)^. However, the small number of studies examining behavioral data points to a lack of strong evidence.

**Table 1.** Effects of tACS on cortico-cortical networks on FC and performance

Study name	Participant numbers	tACS parameters (phase/int/freq/duration)	FC changes (during/post)	Performance Changes (during/post)	Comments
Helfrich et al. 2014	14	in-phase/1 mA/40 Hz/20 min	↑/→	NA/NA	
		anti-phase/1 mA/40 Hz/20 min	↓/→	NA/NA	
Alekseichuk et al. 2017	25	in-phase/2 mA/6 Hz/18 min	NA/→	→ (WM)/NA	
		anti-phase/2 mA/6 Hz/18 min	NA/↓	↓ (WM)/NA	
Tesche et al. 2020	8	anti-phase/1 mA/10 Hz/20 min	NA/→	NA/→ (VA)	
Ahn et al. 2021	13	anti-phase/2 mA/IAF/20 min	NA/↓	NA/NA	
	4	in-phase/4 mA/IAF/20 min	NA/→	NA/NA	
Wei et al. 2021	12	anti-phase/1 mA/6 Hz/15 min	NA/NA	NA/NA	
Fujiyama et al. 2023	18	in-phase/1 mA/20 Hz/15 min	NA/→ (rs-EEG), ↑ (ERP)	→ (RT), → (SRTT)/→ (RT)/→ (SRTT)	Online tACS
	18	in-phase/1 mA/20 Hz/15 min	NA/→ (rs-EEG), → (ERP)	→ (RT), ↑ (SRTT)/→ (RT), ↑ (SRTT)	Offline tACS

The effects of tACS are indicated by the arrow direction for FC (increase: ↑; no change: →; decrease: ↓) or (improvement: ↑; no change: →; decline: ↓).

EEG, electroencephalography; ERP, event-related potential; FC, functional connectivity; IAF, individual alpha frequency; NA, not available; RT, reaction time; SRTT, stop signal reaction time; tACS, transcranial alternating current stimulation; WM, working memory

In this minireview, we initially anticipated that tACS applied to 2 regions could be a useful tool for modulating specific cortico-cortical networks. However, the use of dual-region tACS remains limited. We explored the effects of in-phase and anti-phase tACS on cortico-cortical networks but found no strong evidence that tACS consistently modulates network activity based on the retrieved studies. Recent research suggests that both in-phase and anti-phase tACS can influence FC^23,44,46,47)^ and behavior^44,46)^ in specific cortico-cortical networks. However, due to the small number of studies, the findings do not allow for a definitive conclusion that tACS reliably strengthens or weakens cortico-cortical networks based on the stimulation phase. Therefore, this minireview highlights future directions for rigorously investigating how tACS may modulate cortico-cortical networks, rather than focusing on interpreting the current results. If tACS-induced neural entrainment is effective at the cortico-cortical level, it could serve as a valuable method for selectively enhancing or weakening cortico-cortical networks. This approach may be especially useful for addressing brain disorders associated with dysfunctional cortico-cortical networks, potentially offering broader and more effective outcomes than conventional NIBS, which usually targets individual brain regions.

Most studies have assessed FC before and after the intervention to examine the effects of tACS on specific cortico-cortical networks. However, neural entrainment may be more evident during tACS itself, as animal studies have shown that neural spikes synchronize with the phase of tACS in real time^21,25,26)^. Investigating this online effect is crucial but difficult due to significant artifacts caused by tACS, as the tACS frequency often overlaps with the frequency of interest for FC. A review paper proposed artifact rejection methods to reduce tACS-related artifacts, allowing the study of neural entrainment during stimulation.^48)^ However, FC changes during tACS have not yet been explored using these methods, which are still limited to single brain regions. Despite these advances, artifact rejection remains imperfect, making it challenging to study online tACS effects. Further research is needed to determine whether tACS can reliably alter specific cortico-cortical networks during and after stimulation, as well as isolated regions, through neural entrainment.

MEG studies have shown that specific cortico-cortical networks are crucial for motor and somatosensory performance^1,2,30,38)^. However, the potential of tACS to modulate these networks and affect specific behaviors is still largely unexamined, with only 3 relevant studies identified in the literature search^44–46)^. In comparison, targeted NIBS interventions applied to single brain regions have demonstrated distinct region-specific effects: changes in M1 excitability enhance motor performance^49,50)^, while changes in S1 excitability improve somatosensory performance^51,52)^. These findings emphasize the significant role of excitability changes in individual brain regions on their associated behaviors. At the cortico-cortical level, limited evidence suggests that modifying specific cortico-cortical networks can influence behavior^44,46)^. However, it remains unclear whether targeting individual brain regions or cortico-cortical networks is more effective in improving behavior. Since behaviors are processed by specific cortico-cortical networks, tACS targeting these networks may be more effective in enhancing behavioral outcomes. Future studies should investigate whether targeting individual brain regions or cortico-cortical networks is more successful in driving behavioral changes.

This minireview primarily focused on studies that estimated FC based on EEG sensor data. Accurate FC estimation requires source analysis^6)^, which maps sensor-level signals to cortical sources^53)^. Sensor-level analysis lacks the spatial resolution needed to pinpoint the exact brain regions generating brain oscillations. For example, alpha oscillations recorded at the Cz electrode may not originate directly beneath it but could instead arise from other regions through volume conduction. To overcome these limitations, source analysis is recommended for precise measurement of cortico-cortical interactions. This requires high-density sensor arrays and individualized MRI in EEG studies. Tools like Brainstorm^53)^, FieldTrip^54)^, and EEGLAB^55)^ offer free source analysis methods and support various FC metrics. Among the reviewed studies, the PLV^56)^ and phase lag index^57)^ were the most commonly used FC estimation measures. FC in EEG/MEG studies is generally categorized into 2 types: phase-based and power-based FC^35)^. Since tACS uses alternating current stimulation, phase-based FC is especially useful for studying neural entrainment between regions. By contrast, fMRI may be less suitable for assessing tACS effects on FC, as it detects slower signal fluctuations and relies on power-based FC through envelope signals^6)^.

**Fig. 2.**
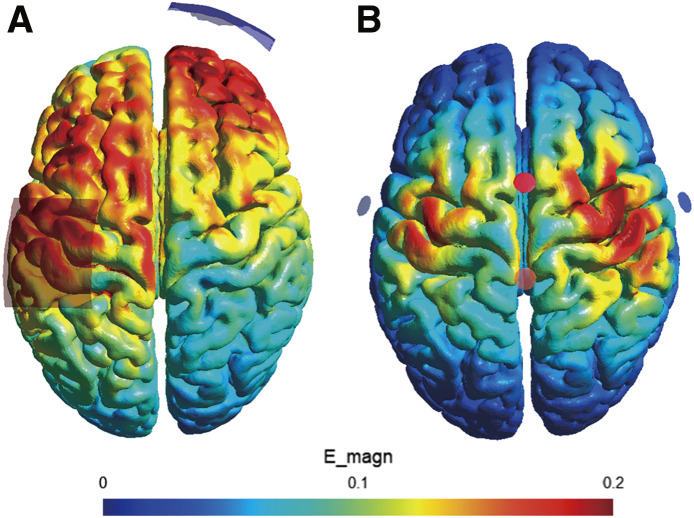
Simulation of electrical field strength using a template MRI (A) Standard tACS montage targeting the left M1, with each electrode measuring 5 cm × 7 cm and an intensity of 1.0 mA. (B) Optimized tACS montage targeting both left and right M1. This optimization identified the optimal electrode placements and intensities to achieve 0.2 V/m at the targeted regions. The configuration used electrodes (diameter = 1 cm) with a maximum intensity of 1 mA. The optimal stimulation placements and intensities were determined as Cz (±1 mA), C3 (±0.79 mA), C4 (±1 mA), and CPz (±0.79 mA) tACS, transcranial alternating current stimulation

When applying tACS to stimulate 2 regions, 4 additional electrodes are usually required. Effective stimulation depends on selecting optimal electrode placements, but this is challenging due to the limited research on targeting 2 brain regions. Furthermore, the strongest electrical field often does not directly align beneath the electrode. For example, we simulated the electrical field distribution using a traditional montage targeting M1 (Fig. 2A). In this setup, one electrode was placed near M1 and the other on the forehead. This montage does not seem to provide focal stimulation of M1, as the electrical field is distributed across a wide range of brain regions. Optimization tools like SimNIBS are therefore crucial. This tool identifies the strongest electrical field based on EEG channel locations^58)^, optimizing the effectiveness of dual-region tACS. For example, we simulated the electrical field distribution targeting the left and right M1 (Fig. 2B). This setup was designed to achieve 0.2 V/m in both M1 regions using 4 electrodes. These simulations emphasize the importance of advanced tools like SimNIBS for optimizing electrode placements and intensities in dual-region tACS, ensuring precise and effective stimulation of target regions.

## Conclusions

This minireview summarized the effects of tACS on specific cortico-cortical networks and related behaviors. Our findings suggest that evidence for changes in FC and behavior resulting from simultaneous tACS targeting 2 regions is inconsistent and based on a limited number of studies. We also highlighted future directions for investigating network modulation to deepen our understanding of cortico-cortical interactions. While traditional NIBS techniques typically target individual brain regions, recent studies indicate that behavior is regulated by interconnected brain regions. Dysfunctional cortico-cortical networks have been associated with conditions like epilepsy^59)^, attention deficit hyperactivity disorder^60)^, and stroke^61)^. If tACS can modulate these networks, it may offer considerable potential for neurorehabilitation. With ongoing advancements in research and technology, targeting cortico-cortical networks with tACS could provide innovative and effective neurorehabilitation strategies, offering new opportunities to improve outcomes across various brain functions.

## Funding

This work was supported by a Research Fellowship for Young Scientists and a grant-in-aid for JSPS Fellows from the Japan Society for the Promotion of Science (Grant No.: 222270200002MY and 23KJ1810).

## Conflicts of Interest

The author declares no conflict of interest.

## Appendix 1

Search: (transcranial alternating current stimulation) AND (functional connectivity) Filters: Humans, English, Adult: 19+ years.
